# Final-year university students’ mental health and access to support as they prepared to graduate

**DOI:** 10.1080/28324765.2023.2252918

**Published:** 2023-09-24

**Authors:** Megan J. Magier, Madelyn Law, Sarah Pennisi, Tanya Martini, Markus J Duncan, Hussain Chattha, Karen A Patte

**Affiliations:** a Faculty of Applied Health Sciences, Brock University, St. Catharines, ON, Canada; b Student Services and Wellness, Niagara College, Welland, ON, Canada; c Department of Psychology, Faculty of Social Sciences. Brock University, St. Catharines, ON, Canada; d Children’s Hospital of Eastern Ontario Research Institute, Ottawa, ON, Canada

**Keywords:** emerging adulthood, transitions, mental health, service use, post-secondary students

## Abstract

Previous research has examined postsecondary student mental health and transitions into university. However, research focused on the transition out of university is lacking. Challenges may be experienced differently among population subgroups. We examined the mental health and support access of university students approaching graduation and differences by various social positions. Survey data were collected from final-year undergraduate students that had registered to graduate at a Canadian university in 2021 and 2022. Chi-square and linear regression models analyzed relationships between sociodemographic characteristics and mental health outcomes. Open-ended questions assessed barriers to accessing support and desired supports. Sexual/gender diverse students reported greater depressive symptoms than cisgender heterosexual students. Students without stressful childhood or current financial situations had lower depression and anxiety scores than their peers that experienced stressful financial situations, respectively. Formal support was more commonly accessed off-campus than on-campus in the past year. Differences in past-year support access were found by gender/sexuality, financial stress, age and race/ethnicity. Availability/scheduling was the most reported barrier to accessing campus-based services. Financial concerns were a common challenge and area for desired support. Implementing developmentally specific mental health support catering to the demands of this life period is necessary.

## 1. Introduction

Previous research has examined university student mental health and transitions into university (Arnett et al., 2014; Grosemans et al., 2020); however, studies focusing on the transition out of university are lacking. Most individuals that pursue postsecondary education will begin and graduate from their programs during the developmental period referred to as emerging adulthood (18–29 years). Arnett proposed emerging adulthood to be a distinct stage between adolescence and adulthood where broad social changes have led to extended periods of finding stable employment and entering adulthood (Arnett, 2015; MacLeod & Brownlie, 2014). While many individuals experience positive transitions, those without sufficient support and resources or with pre-existing mental disorders may struggle. More widespread participation in postsecondary education is occurring for emerging adults, resulting in an increasingly diverse student body (Eisenberg & Lipson, 2019). Thus, new evidence is needed to understand experiences of mental health and support access among contemporary postsecondary student populations as they prepare to graduate.

Postsecondary student mental health has been identified as key public health concern. In the 2018–2019 Healthy Minds Study (*N* = 62,171), approximately 36% of postsecondary students in Canada and the United States reported major or moderate depression, 31% reported an anxiety disorder, and 24% were taking psychiatric medication within the past year (Carver et al., 2015). The high prevalence of mental disorders among students is partially explained by students entering postsecondary education during a time when mental disorders peak. In support, emerging adults report higher rates of depression and anxiety than individuals during other developmental periods (Carver et al., 2015; MacLeod & Brownlie, 2014). The expansion of postsecondary enrollment has also been suggested as contributing to heightened need for campus-based mental health services (Eisenberg & Lipson, 2019).

The high rates of mental disorders and suicidality among emerging adults point to an urgent need to intervene because poor mental health and mental disorders are significant predictors of impaired educational attainment, employment and relationships and increased physical morbidity and early mortality (Arnett et al., 2014; Wiljer et al., 2016). However, emerging adults also have elevated rates of disengagement from mental health services relative to individuals during other developmental periods (Carver et al., 2015; MacLeod & Brownlie, 2014). Generally, informal sources of mental health support (e.g. from social relationships such as friends and family) tend to be sought out over formal support (Arnett, 2007). Formal support refers to resources and services provided by professionals who have appropriate training to provide assessment and treatments (i.e. mental health services and health professionals).

Postsecondary institutions represent key contexts to reach emerging adults. Baik, Larcombe & Brooker (Baik et al., 2019) argue that universities have an ethical responsibility to support and ensure the safety of their students. Improving access to support involves raising awareness about services and ensuring they meet the growing demand of students, reducing stigma and improving students’ mental health literacy (Arnett, 2015). A “whole university” approach has been recommended, which involves going beyond mental health service access to foster a supportive social and educational environment (e.g. changes to syllabi, cultivating supportive interactions with professors and peers) (Baik et al., 2019; Wiljer et al., 2016).

Experiences of postsecondary education vary within student populations and research suggests inequities in mental health and access to support. Postsecondary students identifying as women, lesbian, gay, bisexual, transgender, queer and questioning and two spirit (LGBTQ2S), Black and persons of colour, and students from lower income households have demonstrated elevated risk of mental disorders relative to students identifying as cisgender men, heterosexual, White and from more affluent households, respectively (Chrikov et al., 2020; Hallett et al., 2020; House et al., 2020; Kirmayer et al., 2011; Silver & Roksa, 2017; OECD, 2013). For example, stigma and discrimination contribute to the increased risk of poor mental health that LGBTQ2S individuals experience; heteronormative biases also create barriers for LGBTQ2S individuals in accessing mental health supports (Czeisler et al., 2020; Silver & Roksa, 2017). Recently immigrated and international postsecondary students may also experience unique stressors related to support from family and friends, cultural expectations and language (Chrikov et al., 2020; Hallett et al., 2020). In some cultures, mental disorders are particularly stigmatized, and disclosure of poor mental health and/or mental disorders can be regarded as weakness (Xiong et al., 2020). Living situations and current financial stress have been linked to poor mental health, particularly in international undergraduate students; living on campus may lead to feelings of loneliness and homesickness, whereas feeling financially confident has been associated with positive emotional wellbeing (Chaiton et al., 2023; Wallach et al., 2020). Another population identified as in potential need of additional support is first-generation university students (i.e. students without a parent/guardian that have pursued postsecondary education) (House et al., 2020). Further understanding of disparities in mental health and support access is critical to address barriers and ensure interventions appropriately and effectively met the needs of diverse student populations, particularly those belonging to equity deserving groups.

The purpose of this study was to examine the mental health of final-year university students and the extent to which they accessed informal and formal mental health support as they prepared to graduate. Given the sociocultural and economic factors influencing experiences of university and transitions (Chrikov et al., 2020; Linden & Jurdi-Hage, 2017; MacLeod & Brownlie, 2014; Mori, 2000), and mental health and help-seeking disparities (Barkham et al., 2019; Hyun et al., 2006), a secondary aim was to examine differences in mental health and supports in population subgroups, defined by social positions and identity factors (e.g. gender, affluence). Specific research questions were:

- What was the mental health status (depression, anxiety, perceived stress, psychosocial wellbeing) of final-year undergraduate students as they prepared to graduate?
- How did mental health vary by population subgroups (i.e. age, gender identity, transgender status and sexual orientation, living situation, race/ethnicity, immigrant status, first generation Canadian, parental education/first generation university student, current and childhood financial stress) in a university setting?
- What proportion of final-year undergraduate students received supports for their mental health in the past 12 months, and what types of supports were most common: formal/informal, on campus/off campus?
- Are some population subgroups more likely to access certain types of supports?
- What types of barriers did students face when accessing services in their final year of university?
- What types of supports would students like to receive in their final year?

## 2. Methods

This study used cross-sectional survey data from the *Student Health in Final-year Transitions (SHIFT) Study*. Ethics approval was obtained from Brock University’s Research Ethics Board (#20–271).

### 2.1. Participants

Data were collected in April 2021 (Y1) and 2022 (Y2). In both years, all students who had registered their intent to graduate during the upcoming spring convocation at a public university in southern Ontario, Canada, received an email invitation to participate in the online survey. The university is considered mid-sized relative to other Canadian universities, with a total 2021 and 2022 enrollment of approximately 17,000 undergraduate students across 6 faculties, and classified as comprehensive (i.e. active research and undergraduate, graduate, and professional programs).

Potential participants were deemed eligible if they were enrolled in their final year of their undergraduate program and had submitted their intent to graduate for the upcoming convocation. Ineligible participants *(n* = 127) were automatically redirected to a page informing them of their ineligibility and thanking them for their interest in participating. In Y1, 281 eligible students responded out of a total of 2866 registered graduating students (9.8%); in Y2, 322 eligible students responded out of a total of 3135 registered graduating students (10.3%).

To maximize the sample size, and the ability to examine differences by population subgroups, statistical analyses were conducted to assess whether the 2 years of data collected were similar in order to combine them. Independent samples t-tests were performed to examine whether the mental health outcomes (anxiety, depression, psychosocial wellbeing and perceived stress) significantly differed between students participating in Y1 and Y2 (see Appendix 1). No statistically significant differences were found between students that participated in Y1 and Y2 for anxiety (*t*(562)= −1.4, *p* = 0.2), depression (*t*(562) = 1.5, *p* = 0.1) and psychosocial wellbeing (*t*(562) = 1.6, *p* = 0.1) (see Appendix 1); however, perceived stress was significantly lower in Y1 (*M* = 12.3, SD = 9.7) than Y2 (*M* = 11.1, SD = 15.7), (*t*(562) = 2.9, *p* < 0.01). Based on these analyses, the Y1 and Y2 samples were combined to create a total sample of 587 participants, and study year was entered into multivariable linear regression models to correct for potential differences.

Appendix 2 displays the missing items per variable. Person mean imputation and other missing data strategies were not used due to the nature of the missing data. A complete case analysis was conducted, excluding students who did not respond to all variables, which resulted in a sample of 497. A sensitivity power analysis was conducted in G*Power for analysis of covariance when *n* = 497, α = 0.05, 1- β = 0.80, with 13 predictors controlled for. The model would be able to detect a group difference when there are three categories, with an effect size as small as Cohen’s f = 0.14; based on effect size guidelines (Cage et al., 2021), these analyses would be able to detect small differences between groups as statistically significant.

### 2.2. Data collection

An invitation letter and subsequent follow-up email reminders included a link to the Information and Consent page on Qualtrics. Participants were required to provide consent by clicking a checkbox on the online Information and Consent page before proceeding to the survey. The initial email invitation with a link to the survey hosted on Qualtrics was sent on 19 April 2021, in the first year, and on 18 April 2022, in the second year, with two follow-up reminder emails both years. The surveys remained open for 3-week data collection periods. Participants were able to enter an optional draw to win one of ten $50 gift cards.

### 2.3. Measures

#### 2.3.1. Sociodemographic measures

Sociodemographic variables were chosen based on evidence of mental health disparities in postsecondary students. Students were asked about their age, gender identity, sexual orientation, transgender status, race/ethnicity, employment, living situation, country of birth, parental education level and country of birth, and if they perceived their financial situation to be stressful currently or while growing up. A table of student demographic measures and the full response options can be found in Appendix 3. Low frequencies required some demographic categories to be collapsed for analyses (see Appendix 4).

Age was categorized as 20–23, 24–27 or 28–50, due to the low frequency of older ages. Gender, sexual orientation and transgender status were collapsed into heterosexual cisgender women, heterosexual cisgender men and sexual/gender diverse (i.e. all other combinations and responses, including non-binary, two-spirit, lesbian/gay, bisexual/pansexual, asexual, queer, questioning/unsure, other and prefer not to answer) due to low frequencies within these variables. Race/ethnicity was classified as either White or Black, Indigenous, and persons of colour (BIPOC; including response options: Indigenous peoples of Canada, Indigenous peoples outside of Canada, Arab, Black, Chinese, Filipino, Japanese, Korean, Latin, Central or South America, South Asian, Southeast Asian and West Asian, another response and multiple responses). Current and childhood financial situations were classified as stressful if they responded, “always stressful”, “often stressful” or “sometimes stressful” and not stressful if they responded, “rarely stressful” or “never stressful”. A measure of parental education was used to determine if students were first generation postsecondary students. Parental education was categorized as at least some postsecondary education (including “Completed a college/university degree” and “Completed a graduate/professional program”), high school or less and other (including “I don’t know” and “I prefer not to answer”). Students not born in Canada were classified as Immigrants, students with a parent born outside of Canada were classified as first-generation Canadians, and all other students were classified as other (at least second-generation Canadians). Finally, living status was classified as off-campus with family, off-campus alone or with friends and on-campus.

#### 2.3.2. Mental health measures

Anxiety was measured using the Generalized Anxiety Disorder 7-item scale (GAD-7) (Eisenberg et al., 2012). This scale assesses frequency of anxiety symptoms in the past 2 weeks, such as feeling nervous, anxious or on edge, not being able to stop or control worrying and trouble relaxing. Scores range from 0 to 21, with higher scores indicating greater levels of anxiety symptoms. Response options on a 4-point Likert scale include “Not at all” (0), “Several days” (Arnett et al., 2014), “More than half of the days” (Grosemans et al., 2020) and “Nearly every day” (Arnett, 2015). The GAD-7 was developed as a screening and severity measure and has demonstrated sensitivity and specificity for detecting mild, moderate and clinically significant anxiety symptoms (Crumb et al). Scores were categorized into “no symptoms/mild symptoms” (GAD-7 ≤ 9) and “moderate/severe symptoms” (GAD-7 ≥ 10) (Crumb et al) for descriptive analysis to provide context to scores and treated as continuous for multivariable linear regression analysis. The GAD-7 has demonstrated validity as a measure of anxiety in student populations and has been used in similar studies (Crumb et al). The internal consistency for the current sample is α = 0.992.

Depression was measured using the 9-item Patient Health Questionnaire (PHQ-9) Scale (Chen & Chen, 2010). This scale assesses frequency of depression symptoms over the previous 2 weeks, such as little interest or pleasure in doing things, feeling down, depressed, or hopeless, and feeling tired or having little energy. Response options on a 4-point Likert scale include “Not at all” (0), “Several days” (Arnett et al., 2014), “More than half of the days” (Grosemans et al., 2020) and “Nearly every day” (Arnett, 2015). Sum scores range from 0 to 27, with higher scores indicating greater degrees of depressive symptomatology. Scores were categorized into “no symptoms/mild symptoms” (PHQ-9 ≤ 9) and “moderate/severe symptoms” (PHQ-9 ≥ 10) (Spitzer et al., 2006) for descriptive analysis to provide context to scores and treated as continuous for multivariable linear regression analysis. The PHQ-9 has demonstrated validity in adult primary care settings (Chen & Chen, 2010). The internal consistency for the current sample is α = 0.992.

Stress was measured using the Perceived Stress Scale-4 (PSS-4) which consists of 4-items assessing the degree to which experiences during the past month were perceived as stressful (Mossman et al., 2018). The 4-item scale is demonstrated to be a reliable measure for global perceptions of stress and has demonstrated validity in university populations (Carver et al., 2015). Sum scores range from 0 to 16, with a greater score indicating that the respondent perceives that their demands exceed their ability to cope. Scores were treated as continuous measures, as no clinically relevant cut-off has been established. The internal consistency for the current sample is α = 0.996.

Psychosocial wellbeing was measured on a modified version of Diener’s Flourishing Scale (FS) (Kroenke et al., 2001). The 8-item scale examines self-perceived psychosocial functioning, tapping into life purpose, meaning and satisfaction, engagement and interest in one’s activities, optimism, self-esteem and perceived competence, and relationships (Manea et al., 2012). The original 7-point Likert scale was modified to a 5-point Likert scale from 1 (“Strongly Disagree”) to 5 (“Strongly agree”), removing the “slightly agree” and “slightly disagree” options. This was done to make the measure more appropriate for online survey use, as has been done and validated in previous studies (Cohen, 2022). Scores range from 8 to 40, with higher scores indicating greater levels of flourishing (Kroenke et al., 2001). Scores were treated as continuous measures, as no clinically relevant cut-off has been established. The scale has shown strong psychometric properties across varying age groups (Diener et al., 2009; Warttig et al., 2013). The internal consistency for the current sample is α = 0.997.

#### 2.3.3. Help-seeking measures

Help-seeking measures assessed whether participants had accessed informal (i.e. Roommate, Friend or significant other, Family member, Instructor/Professor, Religious counsellor or other religious contact, Support group, Other nonclinical source) and formal supports (i.e. Mental health related clinician, Other health care professional, Other clinical source) for mental health, on and off campus, in the past year (Hone et al., 2014; Romano et al., 2020), and if they needed mental health support, whether they would know where to access it on or off campus. Students were also asked to indicate the likelihood of accessing different types of programs/resources if they had been available in their final year. Additional measures allowed open-ended responses to questions such as “If you have tried (successful or not) to access formal support for emotional or mental health on campus, please describe any barriers you have encountered” and what types of programs/resources they wished had existed in their final year. These measures were co-developed with our partners from the Student Wellness and Accessibility Center, and from the Co-op, Career and Experiential Education center, to help inform ways to improve services to support graduating final-year students. Additionally, these measures were pilot tested among of group of students, characteristic of our sample to ensure comprehension, comprehensives, relevance and face validity of the measures. A full list of help-seeking measures can be found in Appendix 5.

## 3. Analysis

All statistical analyses were conducted using SAS 9.4. Analyses to examine missing data and adherence to model assumptions were performed. Models were inspected for multicollinearity, and residual Q-Q Plots were used to assess normality of the multivariable models. Sample descriptive statistics (frequencies) were calculated for all sociodemographic measures. For RQ1, descriptive statistics, including frequencies (for bivariate cut-off scores indicating clinically relevant symptoms) and means/medians (for continuous scores), were calculated for depression, anxiety, perceived stress and psychosocial wellbeing. For RQ2, multivariable linear regression models with Bonferroni adjustment for multiple comparisons applied to p-values and 95% confidence intervals (CI) using least squares (LS) mean estimates were conducted assessing student sociodemographic measures as predictors of all four continuous mental health outcomes. Consistent with Keyes’ Dual Continuum Model of complete mental health and similar studies (Howell & Buro, 2015; Keyes, 2005; Wallach et al., 2020), depression and anxiety were presented together as symptoms on the mental illness continuum and perceived stress and psychosocial wellbeing were presented together as measures representing the mental health continuum. A critical p-value of 0.05 was used. Confidence intervals were corrected using Bonferroni adjustments and used to interpret results to reduce the risk of Type 1 error. Additionally, results were adjusted for multiple comparisons, and 2 years of data were combined to maximize the sample size. For RQ3, the proportions of students who received different types of supports (formal, informal, on-campus and off-campus) for their emotional or mental health in the past 12 months were examined. For RQ4, frequency and chi-square analyses were used to examine whether certain populations subgroups were more likely to have accessed different types of supports for their mental health. Finally, for RQ5 and RQ6, barriers students faced to accessing services during their final year of university and the types of supports that they wish existed were analyzed through students’ open-ended responses to these questions. All responses were initially read to become familiar with the data and condensed to create a simplified unit of analysis. Responses were coded posteriori based on words and/or phrases that arose in the data. Similar codes were then grouped into categories. Based on the codes contained in the categories, themes were generated from the data. Codes, categories and themes were re-analyzed and re-categorized until finalized and the most frequently mentioned themes were reported (Popping, 2015).

## 4. Preliminary results

Bivariate Pearson correlation coefficients for the mental health outcomes were conducted (see Appendix 6) which indicated some concern for multicollinearity. To address this, the variance inflation factor (VIF) and tolerance were tested for in all variables. The VIF was close to 1 for all variables, which indicates multicollinearity is not a concern (Oke et al., 2022; Schroeder et al., 1990). The tolerance values were also below 1, indicating no multicollinearity in the variables (Oke et al., 2022; Schroeder et al., 1990). Moreover, depression and anxiety models were not tested in the same model to further eliminate any concern for multicollinearity. Residual Q-Q Plots (see Appendix 7) indicated non-normality within psychosocial wellbeing and perceived stress; however, the tests used are robust to non-normality with a large sufficiently large sample size (Sainani, 2012). LS mean estimates do not require normal distribution for validity (Tellinghuisen, 2008).

Table 1 provides sociodemographic characteristics of the full sample and by study year. Three quarters of the total sample were 20–23 years of age, 59.3% (*n* = 345) were heterosexual cisgender women, and 65.5% *(n* = 341) identified as White. Most participants were born in Canada (79.4%, *n* = 458); about half of the sample had a parent born outside of Canada (48.0%, *n* = 275). The majority of students were living off campus with family (56.4%, *n* = 331) or with friends/roommates (32.7%, *n* = 192). Half of the sample perceived their financial situation to be stressful while growing up, and 73.9% (*n* = 420) perceived their current financial situation to be stressful. Finally, 16.0% (*n* = 91) of students did not have a parent that had completed any postsecondary education.

**Table 1.** Sociodemographic characteristics of graduating university students that participated in year 1 (2021) or year 2 (2022) of the SHIFT study

		Total	Y1	Y2
		N (%)/M (SD)	N (%)/M (SD)	N (%)/M (SD)
Age	20–23	429 (75.0%)	221 (79.5%)	208 (70.7%)
	24–27	90 (15.7%)	31 (11.2%)	59 (20.1%)
	28–50	53 (9.3%)	26 (9.4%)	27 (9.2%)
Gender & Sexual Orientation	Heterosexual cisgender women	345 (59.3%)	177 (63.0%)	168 (55.8%)
	Heterosexual cisgender men	132 (22.7%)	60 (21.4%)	72 (23.9%)
	Sexual/gender diverse^a^	105 (18.0%)	15.7%)	61 (20.3%)
Living Situation	On-campus	12 (2.0%)	3 (1.1%)	9 (3.0%)
	Off-campus with family	331 (56.4%)	185 (65.6%)	146 (47.9%)
	Off-campus alone	40 (6.8%)	15 (5.3%)	25 (8.2%)
	Off-campus with friends/roommates	192 (32.7%)	76 (27.0%)	116 (38.0%)
	Other (no stable housing, prefer not to answer, missing	12 (2.0%)	3 (1.1%)	9 (3.0%)
Race/Ethnicity	White	341 (65.5%)	160 (64.5%)	181 (66.3%)
	BIPOC^b^	180 (34.5%)	88 (35.5%)	92 (33.7%)
Born in Canada	Yes	458 (79.4%)	219 (78.2%)	239 (80.5%)
	No	119 (20.6%)	61 (21.8%)	58 (19.5%)
Parents Born in Canada	Yes	298 (52.0%)	153 (55.4%)	145 (48.8%)
	No	275 (48.0%)	123 (44.6%)	152 (51.2%)
Parental Education	High school or less	91 (16.0%)	51 (18.5%)	40 (13.5%)
	College/university degree	366 (63.8%)	180 (65.2%)	186 (62.6%)
	Graduate/professional degree	85 (14.8%)	43 (15.6%)	42 (14.1%)
	Some post-secondary education	6 (1.0%)	2 (0.7%)	4 (1.3%)
	Other (don’t know, prefer not to say)	25 (4.4%)	0 (0.0%)	25 (8.4%)
Current Financial Situation	Stressful	420 (73.9%)	196 (71.8%)	224 (75.9%)
	Not Stressful	148 (25.1%)	77 (28.2%)	71 (24.1%)
Financial Situation Growing up	Stressful	283 (50.0%)	126 (46.5%)	157 (53.0%)
	Not Stressful	284 (50.0%)	145 (53.5%)	139 (47.0%)
Mental Health Score	Anxiety	9.3 (6.8)	9.7 (6.5)	10.1 (6.0)
	Depression	9.9 (6.2)	9.2 (6.7)	9.5 (6.6)
	Wellbeing	28.6 (9.0)	28.0 (10.1)	29.2 (7.9)
	Perceived Stress	10.9 (3.3)	11 (4.1)	11.3 (3.6)

^a^Sexual/gender diverse includes: Non-Binary, Two-Spirit, Prefer not to Answer, Lesbian/Gay, Bisexual/Pansexual, Asexual, Queer, Questioning/Unsure, Other and Prefer not to Answer.

^b^BIPOC includes: Indigenous peoples of Canada, Indigenous peoples outside of Canada, Arab, Black, Chinese, Filipino, Japanese, Korean, Latin, Central or South America, South Asian, Southeast Asian and West Asian.

## 5. Results

### 5.1. Sociodemographic characteristics and mental health descriptive analysis

**Figure 1.**
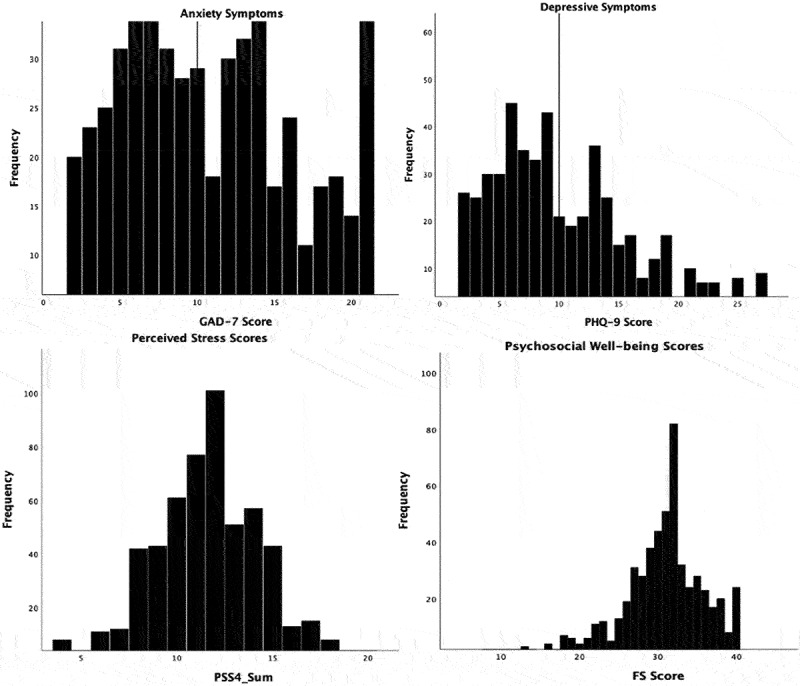
Histograms displaying the distribution of anxiety, depression, perceived stress and psychosocial wellbeing scores in final-year undergraduate students that participated in the SHIFT study.

Note: The lines in the Anxiety and Depression graphs display the threshold for clinically relevant symptoms (GAD-7 ≥ 10, PHQ-9 ≥ 10).

Research question one asked the current mental health status (depression, anxiety, perceived stress, psychosocial wellbeing) of final-year undergraduate students as they prepared to graduate. More than one-third of participants had scores indicative of clinically relevant anxiety (GAD-7 ≥ 10) (41.5%, *n* = 243) and depressive (PHQ-9 ≥ 10) (37.8%, *n* = 221) symptoms. The median and median absolute deviation were 8.0 and 5.0, respectively, for both anxiety and depression scores. Figure 1 displays the distribution of scores for each mental health outcome.

### 5.2. Mental health by subpopulations

Research question two asked how mental health varied by population subgroups in a university setting. Table 2 presents the multivariable linear regression model with adjustment for multiple comparison using LS mean estimates results for depressive and anxiety symptoms by various sociodemographic variables. The adjusted average anxiety score is 1.8 points lower for heterosexual cisgender men (*B* = −1.8, [−3.1, −0.4], t(482) = −2.6, p_adjusted_< .01) than cisgender women. Sexual/gender diverse students reported considerably higher depressive symptoms by 3.4 points (*B* = 3.4, [1.8, 5.0], t(482) = 4.2, p _adjusted_ < .001) than heterosexual cisgender women. The adjusted average score was −2.4 (B = −2.4, [−3.8, −1.0], t(482)= −3.3, p _adjusted_ < .001) and −2.0 (*B* = −2.0, [−3.2, −0.7], t(482)= −3.0, p _adjusted_ < .01) points lower for depression and anxiety, respectively, for students without current stressful financial situations relative to those who reported current stressful financial situations. Similarly, students that were not stressed about their financial situation when growing up had lower adjusted average depression scores (*B* = −1.5, [−2.8, −0.2], t(482)= −2.3, p _adjusted_ < .01) and lower adjusted average anxiety scores (*B* = −1.5, [−2.7, −0.3], t(482)= −2.5, p _adjusted_ < .01) than students reported a stressful financial situation when they were younger. No statistically significant differences were found in depression and anxiety scores based on age category, race/ethnicity, student/parent born in Canada, living situation and parental education level when controlling for the other sociodemographic factors.

**Table 2.** Multivariable linear regression using LS mean estimate models of depression and anxiety symptoms by sociodemographic characteristics among graduating university students in the SHIFT study (2021, 2022)

		Depression		Anxiety	
		Β	95% CI	Β	95% CI
Age	20–23 vs. 24–27	−0.0	[−1.7, 1.6]	1.5	[−0.5, 3.4]
	20–23 vs. 28–50	1.7	[−0.4, 3.8]	1.4	[−0.8, 3.6]
	24–27 vs. 28–50	1.8	[−0.6, 4.2]	0.0	[−1.4, 1.5]
Gender & Sexual Orientation	Heterosexual cisgender men vs. Sexual/Gender diverse	−4.2***	[−6.1, −2.3]	−2.6**	[−4.3, −0.8]
	Heterosexual cisgender men vs. Heterosexual cisgender women	−0.8	[−2.2, 0.7]	−1.8**	[−3.1, −0.4]
	Sexual/Gender diverse vs. Heterosexual cisgender women	3.4***	[1.8, 5.1]	0.9	[−0.6, 2.4]
Race/Ethnicity	BIPOC vs. White	0.4	[−1.2, 2.1]	0.9	[−0.6, 2.4]
Student/ parent born in Canada	First generation Canadian vs. Not born in Canada	0.2	[−1.4, 1.8]	−0.3	[−1.8, 1.1]
	Second+ generation Canadian vs. Not born in Canada	1.2	[−0.8, 3.2]	1.3	[−0.6, 3.1]
	Second+ generation Canadian vs. First generation Canadian	−1.0	[−2.8, 0.7]	−1.6	[−3.2, 0.0]
Parental Education	At least some postsecondary vs. High school or less	−0.3	[−2.0, 1.4]	0.3	[−1.2, 1.9]
	Other vs. High school or less	0.4	[−2.7, 3.5]	0.4	[−2.4, 3.3]
	Other vs. At least some postsecondary	−0.7	[−3.5, 2.1]	−0.1	[−2.7, 2.5]
Current Financial Situation	Not stressful vs. Stressful	−2.4***	[−3.8, −1.0]	−2.0**	[−3.2, −0.7]
Financial Situation Growing up	Not stressful vs. Stressful	−1.5**	[−2.8, −0.2]	−1.5**	[−2.7, −0.3]
Living Situation	On-campus vs. off-campus alone or with roommates	0.8	[−3.2, 4.9]	0.7	[−3.0, 4.4]
	Off-campus with family vs. On-campus	0.5	[−0.7, 1.7]	0.8	[−0.3, 1.9]
	Off-campus with family vs. Off-campus alone or with roommates	0.3	[−3.7, 4.4]	−0.2	[−3.8, 3.5]

Notes: ** = *p* < .01, *** = *p* < .001. Reported p-values adjusted for multiple comparisons. Depression symptoms measured using the PHQ-9. Anxiety symptoms measured using the GAD-7.

Table 3 presents the linear regression model results for psychosocial wellbeing and perceived stress by various sociodemographic variables with adjustment for multiple comparisons using LS mean estimates. The adjusted average psychosocial wellbeing score was substantially lower for sexual/gender diverse students by 4.8 points than heterosexual cisgender women (*B* = −4.8, [−6.9, −2.8], t(482)= −4.7, p _adjusted_ < .001). The adjusted averaged psychosocial wellbeing score was also higher among students who self-reported as BIPOC relative to White students by 3.2 points (*B* = 3.2, [1.1, 5.3], t(482) = 3.0, p _adjusted_ < .01). Students who did not perceive their financial situation to be stressful currently or while growing up reported lower perceived stress than their peers with stressful financial situations by 1.1 and 0.8 points, respectively (*B* = −1.1, [−1.9, −0.3], t(482)= −2.8, p _adjusted_< .01; *B* = −0.8, [−1.5, −0.1], t(482)= −2.3, p _adjusted_< .05). No other included sociodemographic variables had statistically significant associations with psychosocial wellbeing or perceived stress when controlling for other factors in the regression models.

**Table 3.** Multivariable linear regression using LS mean estimate models predicting psychosocial wellbeing and perceived stress by sociodemographic characteristics among graduating university students in the SHIFT study (2021, 2022)

		Psychosocial wellbeing ^a^		Stress ^b^	
		Β	95% CI	Β	95% CI
Age	20–23 vs. 24–27	0.1	[−2.5, 2.8]	0.4	[−0.8, 1.5]
	20–23 vs. 28–50	−0.2	[−3.3, 2.8]	0.4	[−0.9, 1.7]
	24–27 vs. 28–50	0.3	[−1.7, 2.4]	−0.0	[−0.9, 0.9]
Gender & Sexual Orientation	Heterosexual cisgender men vs. Sexual/Gender diverse	4.5**	[2.1, 6.9]	−0.9	[−1.9, 0.1]
	Heterosexual cisgender men vs. Heterosexual cisgender women	−0.4	[−2.2, 1.5]	−0.3	[−1.1, 0.5]
	Sexual/Gender diverse vs. Heterosexual cisgender women	−4.8***	[−6.9, −2.8]	0.7	[−0.2, 1.6]
Race/ Ethnicity	BIPOC vs. White	3.2**	[1.1, 5.3]	0.2	[−0.7, 1.1]
Student/ parent born in Canada	First generation Canadian vs. Not born in Canada	0.1	[−1.9, 2.1]	0.2	[−0.6, 1.1]
	Second+ generation Canadian vs. Not born in Canada	1.0	[−1.5, 3.5]	0.3	[−0.8, 1.4]
	Second+ generation Canadian vs. First generation Canadian	−0.9	[−3.1, 1.3]	−0.1	[−1.0, 0.9]
Parental Education	At least some postsecondary vs. High school or less	0.6	[−1.5, 2.8]	−0.5	[−1.5, 0.4]
	Other vs. High school or less	−0.1	[−4.0, 3.8]	−0.3	[−2.0, 1.4]
	Other vs. At least some postsecondary	0.7	[−2.8, 4.2]	−0.3	[−1.8. 1.3]
Current Financial Situation	Not stressful vs. Stressful	1.0	[−0.7, 2.8]	−1.1**	[−1.9, −0.3]
Financial Situation Growing up	Not stressful vs. Stressful	−0.7	[−2.3, 0.9]	−0.8*	[−1.5, −0.1]
Living Situation	On-campus vs. off-campus alone or with roommates	−0.1	[−5.2, 5.0]	−0.6	[−2.8, 1.6]
	Off-campus with family vs. On-campus	0.1	[−1.4, 1.6]	0.4	[−0.2, 1.1]
	Off-campus with family vs. Off-campus alone or with roommates	−0.2	[−5.3, 4.8]	−1.0	[−3.2, 1.2]

Notes: * = *p* < .05, ** = *p* < .01, *** = *p* < .001. Reported p-values adjusted for multiple comparisons.

^a^Psychosocial Wellbeing measured by the Flourishing Scale; higher scores represent greater psychosocial wellbeing; scores range from 8–56.

^b^Perceived Stress Scale; higher scores correlate to more stress; scores range from 0–16.

### 5.3. Mental health support access

**Figure 2.**
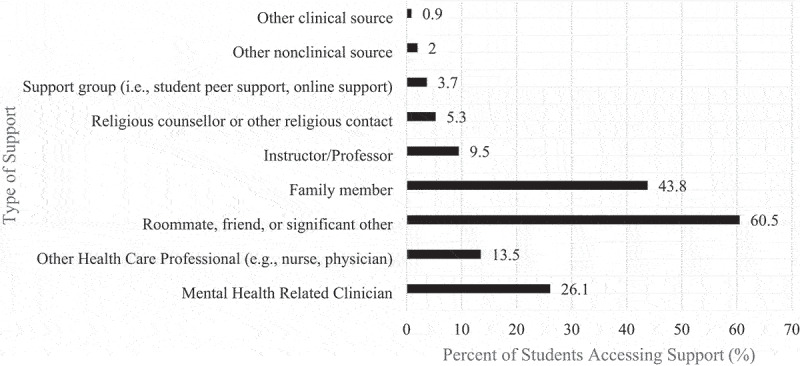
Types of mental health support accessed by participating university students in the final year of their undergraduate program (2021, 2022; *N* = 587).

Research question three asked what proportion of final-year undergraduate students received supports for their mental health in the past 12 months and what types of supports were most common. Figure 2 displays the types of mental health support accessed by participating undergraduate students in the final year of their program. More students reported accessing informal supports (70.9% *n* = 416) than formal (32.5%, *n* = 190). Seeking support from a roommate, friend or significant other—a form of informal support—was the most selected type of support that students accessed (60.5%, *n* = 355). For formal supports, more students reported accessing services off campus (17.4%, *n* = 102) than on campus (13.9%, *n* = 81), although over half of participants agreed that, if they needed to, they would know where to access support on campus for their mental health (64.3%, *n* = 377). Moreover, 6.9% (*n* = 40) of students reported seeking formal support both on and off campus.

Research question four aimed to examine if certain population subgroups were more or less likely to access certain types of supports. Table 4 presents frequency and chi-square results for the different subpopulations and support access. Students that were aged 20–23 years were the age group most likely to have accessed informal support in the past year (70.9%, *n* = 304), while students 24–27 years of age were most likely to have accessed formal support in the past year (43.3%, *n* = 39) and students 28–50 years were most likely to report they would know where to access support on campus if they felt they needed to (40.0%, *n* = 21). By gender and sexuality, heterosexual cis-women were marginally more likely to have sought out informal support in the past year (72.5%, *n* = 250) and gender/sexual diverse students were most likely to have sought formal support and to know where to access support (45.7%, *n* = 48; 39.0%, *n* = 41, respectively). More BIPOC students indicated seeking formal support in the past year (35.6%, *n* = 64 vs. 28.7%, *n* = 98), while more White students accessed informal support (72.5%, *n* = 240 vs.70.0%, *n* = 126). No difference in knowing where to access support on campus was found by race/ethnicity. Students not born in Canada were marginally more likely to report accessing informal supports (73.4%, *n* = 196) than first generation Canadian students (72.9%, *n* = 137). Students with clinically relevant depression and anxiety symptoms were more likely to report knowing where to access support (36.5%, *n* = 81; 35.5%, *n* = 91, respectively) and to have accessed formal and informal support, than their peers without clinically relevant symptoms. Students living off campus alone or with friends were more likely to report knowing where to access support (37.1%, *n* = 86) than students living off-campus with family (31.0%, *n* = 102). A higher proportion of students that perceived their financial situation to be stressful currently or while growing up reported knowing where to access support, and having accessed formal and informal support, than students that did not experience stressful financial situations at either time point.

**Table 4.** Frequency and Chi-square results for knowing where to access support, and access of formal and informal support in the past year, by sociodemographic characteristics among graduating university students in the SHIFT study

		Know where to access N (%)	Chi-square (df) p-value	Formal support N (%)	Chi-square (df) p-value	Informal support N (%)	Chi-square (df) p-value
Age	20–23 (*N* = 429)	138 (32.2)	14.5 Carver et al. (2015) 0.025	129 (30.1)	121.0 Carver et al. (2015) <.001	304 (70.9)	115.3 Carver et al. (2015) <.001
	24–27 (*N* = 90)	33 (36.7)		39 (43.3)		63 (70.0)	
	28–50 (*N* = 53)	21 (40.0)		18 (34.0)		39 (73.6)	
Gender & Sexual Orientation	Heterosexual cisgender women (*N* = 345)	110 (32.0)	41.0 Carver et al. (2015) <.001	107 (31.0)	365.6 Carver et al. (2015) <.001	250 (72.5)	352.3 Carver et al. (2015) <.001
	Heterosexual cisgender men (*N* = 132)	42 (31.8)		34 (26.0)		89 (67.4)	
	Sexual/Gender diverse ^a^ (*N* = 105)	41 (39.0)		48 (45.7)		74 (70.5)	
Living Situation	On-campus (*N* = 12)	4 (33.3)	9.96 Carver et al. (2015) 0.126	5 (41.7)	5.76 Arnett (2015) 0.123	12 (100.0)	3.45 Arnett (2015) 0.326
	Off-campus with family (*N* = 331)	102 (31.0)		118 (35.6)		244 (73.7)	
	Off-campus alone or with friends (*N* = 232)	86 (37.1)		64 (27.6)		157 (67.7)	
Race/Ethnicity	White (*N* = 341)	117 (34.3)	1.7 MacLeod and Brownlie (2014) 0.785	98 (28.7)	30.7 MacLeod and Brownlie (2014) <.001	240 (72.5)	24.7 MacLeod and Brownlie (2014) <.001
	BIPOC ^b^ (*N* = 180)	58 (32.2)		64 (35.6)		126 (70.0)	
Student/parent born in Canada	No (*N* = 267)	91 (34.1)	67.6 Carver et al. (2015) <.001	85 (31.8)	1.66 Arnett (2015) 0.647	196 (73.4)	12.6 Arnett (2015) 0.006
	First generation Canadian (*N* = 188)	60 (31.9)		58 (30.9)		137 (72.9)	
	Second+ generation Canadian (*N* = 118)	42 (35.6)		44 (37.3)		78 (66.1)	
Parental Education	High school or less (*N* = 91)	41 (45.1)	16.4 Wiljer et al. (2016) 0.037	28 (30.8)	3.91 MacLeod and Brownlie (2014) 0.418	65 (71.4)	2.3 MacLeod and Brownlie (2014) 0.680
	At least some postsecondary (*N* = 457)	144 (31.5)		151 (33.0)		326 (71.3)	
	Other (*N* = 25)	8 (32.0)		8 (32.0)		20 (80.0)	
Current Financial Situation	Stressful (*N* = 420)	155 (37.0)	51.7 MacLeod and Brownlie (2014) <.001	145 (34.5)	93.2 MacLeod and Brownlie (2014) <.001	309 (73.6)	100.1 MacLeod and Brownlie (2014) <.001
	Not Stressful (*N* = 148)	37 (25.0)		42 (28.4)		99 (66.9)	
Financial Situation Growing up	Stressful (*N* = 283)	103 (36.4)	44.3 MacLeod and Brownlie (2014) <.001	104 (36.7)	90.1 MacLeod and Brownlie (2014) <.001	206 (72.8)	92.2 MacLeod and Brownlie (2014) <.001
	Not Stressful (*N* = 284)	88 (31.0)		82 (28.9)		201 (70.8)	
Depression	Yes (PHQ-9 ≥ 10) (*N* = 222)	81 (36.5)	70.5 MacLeod and Brownlie (2014) <.001	90 (41.0)	449.9 MacLeod and Brownlie (2014) <.001	177 (80.0)	453.4 MacLeod and Brownlie (2014) <.001
	No (PHQ-9 < 10) (*N* = 361)	107 (29.6)		100 (27.8)		237 (66.0)	
Anxiety	Yes (GAD-7 ≥ 10) (*N* = 256)	91 (35.5)	59.5 MacLeod and Brownlie (2014) <.001	102 (40.0)	450.6 MacLeod and Brownlie (2014) <.001	203 (79.3)	455.4 MacLeod and Brownlie (2014) <.001
	No (GAD-7 < 10) (*N* = 327)	101 (31.0)		88 (27.0)		211 (64.5)	

^a^Gender/Sexual minority includes: Non-Binary, Two-Spirit, Prefer not to Answer, Lesbian/Gay, Bisexual/Pansexual, Asexual, Queer, Questioning/Unsure, Other and Prefer not to Answer.

^b^BIPOC category includes: Indigenous peoples of Canada, Indigenous peoples outside of Canada, Arab, Black, Chinese, Filipino, Japanese, Korean, Latin, Central or South America, South Asian, Southeast Asian and West Asian.

Research question five aimed to explore the types of barriers that students faced when accessing services in their final year of university. For the open-ended responses, 135 students responded to the question regarding types of barriers they have faced when accessing services in their final year of university. The top three barriers most frequently reported for accessing mental health services on campus were a lack of availability in scheduling appointments (e.g. scheduling conflicts) (*n* = 25), long wait times (*n* = 13), and perceptions that the services were not helpful (*n* = 9).

Research question six asked about the types of supports students would like to receive in their final year. From a list of various different types of supports, students were frequently reported being likely to access supports that informed them about how to manage finances (i.e. student loans, debt, other budgeting) (74.2%, *n* = 190), how to get supplemental health insurance (e.g. dental, prescriptions) (69.5%, *n* = 178) and how to develop healthy physical activity, eating and other habits (68.4%, *n* = 175), if they had been available to them in their final year. When asked in an open-ended question if there were any additional supports that they wished had existed, the most common responses by students were career-based supports (e.g. career planning, resume building) (*n* = 16) and financial education (e.g. doing taxes, budgeting, finding insurance) (*n* = 5), out of the 56 that responded to this question.

## 6. Discussion

This study provides contemporary evidence on the mental health and support access of final-year undergraduate students at a mid-sized Canadian university as they prepared to graduate. The average depression and anxiety symptom scores for students fell right below the thresholds that indicate clinically relevant symptomatology, and 42% and 38% of students had scores above these thresholds for depression and anxiety, respectively. Of the sociodemographic variables considered, gender, sexuality and financial stress emerged as factors independently linked to final-year student mental health outcomes. Results highlighted the importance of financial stress to graduating student mental health and mental health inequities by gender/sexuality.

Results align with previous literature which estimates around 40% of emerging adults (age 18–29 years old) experience a mental disorder, higher than any other age group (45). The Healthy Minds Study of post-secondary students found prevalence rates comparable to our sample; 41% screened positive for clinically relevant depressive symptoms and 34% screened positive for anxiety (Carver et al., 2015). The large proportion of graduating students with subthreshold depression and anxiety symptoms is concerning. Upon graduation, students lose access to campus-based mental health services while simultaneously encountering many new stressors that arise from navigating a key life transition. Strategies to prevent symptoms from increasing to potentially clinical levels and to support students over the transition are needed, including bridging access to services in the community. Further prospective research following students postgraduation is also necessary to determine predictors of successful transitions.

### 6.1. Sexual and gender diversity and mental health

Our results also point to population subgroups that may warrant tailored approaches. Sexual and gender diverse students reported substantially higher depressive symptom scores and considerably lower psychosocial wellbeing, although no differences were reported for perceived stress, relative to their heterosexual cisgender peers. Sexual and gender diverse students were also more likely to have accessed formal support in the past year than their heterosexual cisgender peers. We are not able to determine from this study whether the differences in depressive symptoms were connected to those in support access. Relatedly, we do not know whether there was a difference in the need for support and if this was associated with the likelihood of having accessed it. Future research should explore whether there are unmet needs for mental health support among students by sexuality and gendered subgroups.

These findings are consistent with literature displaying the mental health inequities in sexual and gender diverse populations (Jenkins et al., 2022). LGBTQ2S+ individuals often describe experiences of discrimination as contributing to poorer mental health and wellbeing (Toomey et al., 2010; Wickrama et al., 2005). Compared to heterosexual cisgendered individuals, sexual and gender diverse populations’ experiences of stress are more often chronic, socially based and additive to the ordinary stressors that are experienced by all individuals (Meyer, 2003). Often, sexual and gender diverse individual’s experiences are related to stable underlying social and cultural structures that are beyond the individual’s control (Meyer, 2003). These stressors interact and add up to contribute to a greater risk of negative health for sexual and gender diverse populations. Preparing services to sensitively interact with the unique experiences of gender and sexually diverse emerging adults is important. Providing adequate training and hiring LGBTQ2S+ staff may help to reduce bias and increase knowledge surrounding specific LGBTQ2S+ identities while creating culturally safe environments that effectively address their needs (Czeisler et al., 2020).

Heterosexual cisgender men reported slightly lower anxiety symptoms in comparison to heterosexual cisgender women; however, no differences were reported with respect to depression. Previous literature indicates women are two to three times more likely than men to suffer from anxiety (Oke et al., 2022). Heterosexual cisgender men were also least likely to report having accessed formal or informal support, or to know where to access support if they needed to, while cisgender women were most likely to have accessed informal support. This aligns with previous research that has demonstrated that women tend to seek help for their mental health more often and have more social supports than men (Doherty & Kartalova O’Doherty, 2010; Turner, 2023). While well documented in the literature, it is not clear whether this disparity is due to masculine gender roles and norms that might contribute to men underreporting mental health concerns, or if they truly experience lower anxiety symptoms; masculinity expectations are believed to contribute to lower help seeking behaviours (Doherty & Kartalova O’Doherty, 2010).

### 6.2. Financial stress and mental health

Results highlighted the importance of financial stress for graduating student mental health. Current and childhood financial stress had consistent and independent effects across all four mental health outcomes. As per previous research (Hallett et al., 2020; Tellinghuisen, 2008), students who reported that their financial situations currently and while growing up were not stressful had lower depression and anxiety symptom scores and perceived stress scores than their peers that perceived their financial situation to be stressful. Current and childhood financial stress were also linked to knowing where to access support on campus and having accessed formal and informal support within their final undergraduate degree year. Considering these students are more susceptible to poor mental health outcomes, it is a positive sign that they are accessing support. Meeting students with affordable counselling and other supports that help reduce financial burden may make a substantial impact for graduating students (Guan et al., 2022). Low-income students face unique challenges. Those experiencing financial hardship are more prone to be exposed to unhealthy lifestyles, worse living situations and economic uncertainty (Guan et al., 2022; Jin et al., 2020). Indeed, students in this study reported wanting programs to help them be financially responsible, acquire health insurance and develop positive lifestyle habits. Students that must work while taking classes often express greater psychological stress and a reduced sense of belonging to their institution (Kreniske et al., 2022), which has been associated with many negative mental health outcomes, including anxiety and depression (Fink, 2014; Richardson et al., 2017). Given the cross-sectional design, we cannot determine the temporality of results; however, some studies suggest those with pre-existing mental health concerns are more likely to experience a worse financial situation and more financial stress (Arnett et al., 2014; Wickrama et al., 2005).

Over 70% of students reported stress regarding their current finances, which was adversely associated with their mental health. Support for the mental health of final-year graduating students needs to extend beyond mental health services and should seek to address socioeconomic and other key risk and/or causal factors. Emerging adults that have more practical supports available (i.e. financial supports, transportation, etc.) often see increased success during this transition, and stress overall is decreased for students when resources are available (Arnett, 2015). Interestingly, a UK rapid review found little evidence of association between postsecondary student debt and mental health, whereas subjective measures of financial stress, worry or concern were more consistently associated with worse mental health outcomes, consistent with the current results (McCloud & Bann, 2019). Likewise, individuals who expect financial stress based on their experiences, aspirations and perceptions of their ability to manage finances are more likely to experience depression (Arnett et al., 2014; Toomey et al., 2010). Thus, increasing financial literacy and financial decision-making capability (e.g. budgeting, taxes) and self-efficacy may buffer psychological distress for students (Ryu & Fan, 2022). Financial education was also commonly reported when students were asked if there were any additional supports they wish existed during their final year.

Experiencing financial stress while growing up was also associated with poorer mental health outcomes while controlling for current financial stress. Results add to literature demonstrating the potential life-long impacts of childhood financial stress (Guan et al., 2022). Young adults that experienced financial stress in childhood are at risk of decreased financial and mental wellbeing; long-term perceived financial strain has been linked to negative health outcomes (i.e. depression) later in life (Domènech-Abella et al., 2021; Luo & Waite, 2005). Similarly, students who report growing up in a low-income family typically report higher depression, anxiety and suicidal thoughts (Eisenberg et al., 2007). Hypothesized mechanisms for the independent connection between childhood financial stress and poor mental health outcomes include lower family income during childhood being related to poorer cognitive and emotional development and exposure to more stressful life events; parents may be less able to provide a stable, secure and supportive base for their children growing up (Domènech-Abella et al., 2021; Luo & Waite, 2005; Stansfeld et al., 2008).

### 6.3. Race and mental health

Some differences were also found by race. BIPOC students had higher psychosocial wellbeing and were more likely to indicate seeking formal support in the last year and to know where to access supports on campus relative to White students, but no difference was found in the other mental health outcomes. Further research should examine if differences also exist in retention; overall, emerging adults experience the highest drop-out rates from mental health services (Linden & Jurdi-Hage, 2017), and previous research suggests BIPOC populations are more likely to drop out of treatment, due to stigma, mistrust and feeling culturally misunderstood (Fripp & Carlson, 2017; Gondolf & Williams, 2001; National Alliance on Mental Illness, 2023; Wierzbicki & Pekarik, 1993). Increasing BIPOC representation of support staff and providing cultural competency and anti-racism training may improve the delivery of care for these students (Fripp & Carlson, 2017). Moreover, differences in cultural expectations for the transition to adulthood exist. For example, BIPOC populations tend to express family support (i.e. taking care of children, being able to run and financially support a household) as a marker for reaching adulthood, suggesting greater endorsement of collectivist values, whereas White populations tend to express more individualistic values as a marker for reaching adulthood (i.e. being financially able to support themselves) (Arnett, 2003; Syed & Race, 2013). As differences in cultural expectations upon graduation exist, services and supports that are culturally relevant to support emerging adults during this transition are important (Gondolf & Williams, 2001, Lindsey et al., 2013).

Some American studies also reported higher levels of positive mental health in Black adults (25–74 years) in comparison to white adults (Keyes, 2007; Ryff et al., 2003). Actively coping with adverse circumstances (e.g. discrimination) may be linked to building higher resilience, flourishing, wellbeing and better mental health outcomes (Keyes, 2009; Krieger, 1990). However, emerging research specific to postsecondary BIPOC student populations has suggested an increase in anxiety (Coakley et al., 2021). Research exploring the reasons contributing to the differences in wellbeing and resilience in BIPOC and White samples that are specific to emerging adults is still needed. Further, research comparing the mental health of BIPOC student populations compared with those not in postsecondary is needed to assist in understanding the conflicting evidence that exists.

### 6.4. Subpopulations and mental health

No differences were found in mental health outcomes by whether students or their parents were born in Canada, living situation, parental education level and age range when controlling for the other sociodemographic variables. In contrast, previous research has indicated that younger postsecondary students more frequently report symptoms consistent with negative mental health than their older counterparts (Pancer et al., 2000; Paul & Brier, 2001; Wallach et al., 2020). Further, living on campus compared to off campus has been associated with lower anxiety in postsecondary students (Eisenberg et al., 2013; Jenkins et al., 2022). Immigrant and first-generation postsecondary students have been found to be more likely to experience anxiety and depression, and a lower sense of belonging, contributing to poorer overall health and wellbeing (Kreniske et al., 2022). Controlling for the other sociodemographic variables (e.g. financial stress) in this study may have contributed to the lack of differences found in factors that have linked to mental health disparities in the previous research.

### 6.5. Accessing mental health support

Students with potentially clinically relevant depression and anxiety symptoms appear to be accessing informal support more than formal supports. While it is not surprising that informal support is more commonly accessed, results suggest that some individuals in need of formal support may not be accessing services. Students may reach out to family and peers over formal supports given perceptions that most emerging adults “have it all together” and that experiencing mental health difficulties or help-seeking contradict this image (Eisenberg et al., 2012). While having a strong support system is valuable and may offer a less expensive and more socially acceptable alternative, there is still concern regarding students’ mental health literacy; that is, whether they are able to recognize the severity of their symptoms and when formal support is necessary (Jorm, 2012). Previous literature has suggested that emerging adults may not have the financial means to seek formal support; thus, reaching out to informal supports or online resources as a means of mental health treatment has become popular (Arnett, 2003; National Alliance on Mental Illness, 2023). Although free services are available on campus to all students, addressing the barriers to accessing these services that students brought forward such as scheduling conflicts, long wait times and perceptions that services would not be helpful, may be beneficial to increase uptake (Mori, 2000). One approach that has seen success is the Stepped Care Model 2.0, which re-distributes the existing mental health services in a way that maximizes their effectiveness (Stepped care 2.0, 2022). The most effective yet least resource-intensive resources are delivered first to minimize the wait for immediate services, then if necessary, more resource-intensive treatments can be provided depending on the level of care needed for each student (Stepped care 2.0, 2022). Most individuals report that low-intensity services met at least some of their needs; saving more intensive services for those that need them most (Stepped care 2.0, 2022).

Despite widespread mental health campaigns, more than one-third of final-year students reported not knowing where to access mental health support on campus if they needed it. Students with clinically relevant depression and anxiety scores were more likely to know where to access support if needed, as were gender and sexual diverse students and those with financial stress. Differences by subgroups potentially reflect whether they have accessed or tried to access supports. Peer-led mental health organizations have been shown to raise awareness and help-seeking behaviours among postsecondary students (Hadlaczky et al., 2014; Kirsch et al., 2014; Sontag-Padilla et al., 2018). Peer-led programs show promising results in spotting students of concern and directing them to appropriate resources while potentially mitigating more serious and costly strains on campus mental health services later (Kirsch et al., 2014).

## 7. Strengths and limitations

This study adds to the literature by exploring the understudied population of emerging adults graduating from postsecondary education and their mental health supports. A strength of this study is the use of well-validated mental health scales; however, these measures are not diagnostic, and experiencing depression and/or anxiety symptoms is not a proxy for having a mental disorder. Important differences by SES may exist, but there are challenges in reliable and valid measures of SES among younger populations (Aarø et al., 2009; Currie et al., 1997). The small sample size warrants consideration as it limited the analysis that could be conducted; for example, intersectionality could not be considered, which would have allowed for an exploration how students’ intersecting identities interact and contribute to their experiences. Further, collapsing measurement categories may have masked important differences in sub-populations. Future research is needed to examine differences across and within sexual and gender diverse and BIPOC populations.

The online self-report survey subjects the results to potential recall and social desirability biases, which may lead to potential underreporting of mental health symptoms and use of services (Drapeau et al., 2011). The low response rates limit external validity of the study; results may not generalize to all graduating university students and should be considered exploratory. Relatedly, selection bias warrants consideration as those with more severe mental health concerns may be less likely to participate. Alternatively, those that participated may have had a pre-existing concern they wanted to discuss. However, the survey maintained anonymity; personal identifiers were not connected with participant responses. Data collection also took place after most course work was completed, and participants were assured that completion and/or noncompletion of the survey would not affect their academic standing or ability to graduate. Lastly, the use of a complete case analysis has limitations as it increases loss of information in the data set and may increase type II error.

## 8. Conclusion

Gender, sexuality, and current and childhood financial stress emerged as the factors independently linked to final-year postsecondary student psychosocial wellbeing, depression and anxiety of the sociodemographic variables considered. Results point to population subgroups that may warrant targeted mental health interventions during postsecondary degrees and to promote positive transitions upon graduation. This study also supports the value of upstream approaches to supporting postsecondary student mental health, given the high prevalence of current and former financial stress and associated risk for poor mental health outcomes. Developing interventions that are tailored to the realities of students in this life stage is imperative. It may be beneficial to provide practical supports such as career-related and financial education resources to improve student mental health. Continued efforts to increase awareness of mental health services on and off campus would also appear to be worthwhile.

## Data Availability

The data that support the findings of this study are available from the corresponding author, [MM] upon reasonable request.

## Appendix 1. Independent T-test results for the mental health outcome measures in Y andY 2. of the SHIFT study

**Table:**

	Anxiety		Depression		Perceived Stress		Psychosocial Wellbeing	
	2022	2021	2022	2021	2022	2021	2022	2021
Mean	17.1	16.2	18.5	17.6	12.0	11.2	29.2	28.0
Standard Deviation	41.5	53.3	51.7	65.4	9.7	15.8	58.9	101.7
P(T≤t) two-tail	−1.4		1.5		2.9*		1.6	
t Critical two-tail	2.0		2.0		2.0		2.0	

*Significant at the *p* < 0.05 level (2-tailed).

## Appendix 2. Frequency statistics for participant missing data on each variable in Y 1. and Y 2. of the SHIFT study

**Table:**

Variable	N (%)
Age	15 (2.6)
Gender & Sexual Orientation	1 (0.2)
Employed During School Year	2 (0.3)
Living Situation^a^	12 (2.0)
Race/Ethnicity	66 (11.2)
Born in Canada	7 (1.2)
Parents Born in Canada	11 (1.9)
Parental Education	14 (2.4)
Current Financial Situation	19 (3.2)
Financial Situation Growing up	20 (3.4)
Anxiety^b^	27 (4.6)
Depression^b^	28 (4.8)
Perceived Stress^b^	33 (5.6)
Psychosocial Wellbeing^b^	34 (5.8)

^a^Includes: Other (no stable housing, prefer not to answer) or Missing.

^b^Includes: missing all items on the scale.

## Appendix 3. Student demographic measures from Y 1. and Y 2. of the SHIFT survey

**Table:**

Question	Response Option
“How old are you?”	Open-ended response
“What is your gender identity?”	“Woman”, “Non-binary”, “Two-Spirit”, “Man” and “I prefer not to answer”
“Are you someone with trans experience (meaning that your gender identity does not align with your sex assigned at birth)?”	“Yes”, “No” and “I prefer not to answer”
“What is your sexual orientation?”	“Heterosexual”, “Lesbian/Gay”, “Bisexual/Pansexual”, “Asexual”, “Queer”, “Questioning/Unsure”, “Other. Please specify”: and “I prefer not to answer”
“Where do you currently live?”	“University residence”, Other on-campus housing”, “Off-campus with family (e.g. parents, spouse/partner, children)”, “Off-campus alone”, “Off campus with friends or roommates”, “I do not have stable housing (e.g. couch-surfing, living in a vehicle, facing eviction)” and “I prefer not to answer”
“Please indicate how you self-identify. If you are of mixed-descent, please select all that apply”	“Indigenous peoples of Canada (First Nations, Métis, Inuit)”, “Indigenous (outside of Canada)”, “Arab”, “Black”, “Chinese (including Mainland China, Hong Kong, Macau and Taiwan)”, “Filipino”, “Japanese”, “Korean”, “Latin, Central, or South American (e.g. Brazilian, Chilean, Columbian, Mexican)”, “South Asian (e.g. Indian, Pakistani, Sri Lankan, etc.)”, “Southeast Asian (e.g. Cambodian, Indonesian, Laotian Vietnamese, etc.)”, “West Asian (e.g. Afghan, Iranian, Syrian, etc.), “White” and “If none of the above, please specify”
“Were you born in Canada?”	“Yes” and “No”
“What is the highest level of formal education obtained by your parent(s)/guardian(s)?”	“High school or less”, “Completed a college program (e.g. diploma, certificate, apprenticeship)”, “Completed a university degree (e.g. Bachelor)”, “Completed a graduate or professional degree (e.g. Master’s, PhD, Law, Medicine)”, “I don’t know” and “I prefer not to answer”
“How would you describe your current financial situation?”	“Always stressful”, “Sometimes stressful”, “Rarely stressful”, “Never stressful”
“How would you describe your financial situation while growing up?”	“Always stressful”, “Sometimes stressful”, “Rarely stressful”, “Never stressful”

## Appendix 4. Full sociodemographic characteristic breakdown of graduating university students that participated in Year 1 or Year 2 of the SHIFT study

**Table:**

Variable		N (%)
Age	20–27	525 (91.8)
	28–35	25 (4.4)
	36–42	12 (2.1)
	43–50	10 (1.7)
Gender	Women	420 (72.0)
	Men	154 (26.4)
	Non-Binary	6 (1.0)
	Two-Spirit	1 (0.2)
	Prefer Not to Answer	2 (0.3)
Trans Experience	Yes	12 (2.1)
	No	566 (97.1)
	Prefer Not to Answer	5 (0.9)
Sexual Orientation	Heterosexual	483 (82.8)
	Lesbian/Gay	12 (2.1)
	Bisexual/Pansexual	51 (8.7)
	Asexual	6 (1.0)
	Queer	2 (0.3)
	Questioning/Unsure	18 (3.1)
	Other	4 (0.7)
	Prefer Not to Answer	7 (1.2)
Living Situation	University Residence	10 (1.7)
	Other on-campus housing	2 (0.3)
	Off-campus with family	331 (57.4)
	Off-campus alone	40 (6.9)
	Off-campus with friends or roommates	192 (33.3)
	I do not have stable housing	1 (0.2)
	Prefer not to answer	1 (0.2)
Race/Ethnicity	Indigenous peoples of Canada	14 (2.2)
	Indigenous (outside of Canada)	2 (0.3)
	Arab	12 (1.9)
	Black	35 (5.6)
	Chinese	33 (5.3)
	Filipino	10 (1.6)
	Japanese	3 (0.5)
	Korean	5 (0.8)
	Latin, Central, or South America	17 (2.7)
	South Asian	75 (11.9)
	Southeast Asian	15 (2.4)
	West Asian	8 (1.3)
	White	380 (60.6)
	Other	18 (2.9)
Born in Canada	Yes	458 (79.4)
	No	119 (20.6)
	Prefer not to Answer	0 (0.0)
Parents Born in Canada	Yes	298 (52.0)
	No	275 (48.1)
	Prefer not to Answer	0 (0.0)
Parental Education	High school or less	91 (15.9)
	Completed a college program	155 (27.1)
	Completed a university degree	211 (36.8)
	Completed a graduate or professional degree	85 (14.8)
	I don’t know	5 (0.9)
	Prefer not to Answer	1 (0.2)
	Some post-secondary education but no completed degree	25 (4.4)
Current Financial Situation	Always Stressful	63 (11.1)
	Often Stressful	110 (19.3)
	Sometimes Stressful	244 (42.8)
	Rarely Stressful	107 (18.8)
	Never Stressful	41 (7.2)
	Prefer not to Answer	5 (0.9)
**Financial Situation Growing Up**	Always Stressful	50 (8.7)
	Often Stressful	95 (16.6)
	Sometimes Stressful	138 (24.1)
	Rarely Stressful	172 (30.0)
	Never Stressful	112 (19.5)
	Prefer not to Answer	6 (0.9)

## Appendix 5. Student help-seeking measures from Y 1. and Y 2. of the SHIFT survey

**Table:**

Question	Response
“In the past 12 months, have you received support for your mental or emotional health from any of the following sources? (select all that apply)”	“Mental health related clinician (e.g. psychologist, counsellor, psychotherapist, or psychiatrist)”, “Other health care professional (e.g. nurse, physician)”, “Roommate, friend, or significant other”, “Family member”, “Instructor/Professor”, “Religious counsellor or other religious contact”, “Support group (e.g. student peer support, online support group)”, “Other nonclinical source (please specify)”, “Other clinical source (please specify)”, and “No, none of these”
“Have you accessed formal support for your emotional or mental health (e.g. individual or group counselling) on campus/online through Brock University? (Check all that apply)”	“Yes, within the current school year”, “Yes, prior to the current school year”, “No, I have never accessed campus-based resources/services for mental health”
“Have you accessed formal support for your emotional or mental health (e.g. individual or group counselling) off campus/online outside of Brock University? (Check all that apply)”	Yes, in the current school year”, “Yes, since starting at Brock University”, “Yes, prior to starting at Brock University”, “No, I have never accessed off-campus mental health services”
“How much do you agree with the following statements? If I currently needed to seek professional help for my mental or emotional health… “	“I would know where to access campus-based resources (online or in person)”, “I would know where to access off-campus resources (online or in person outside of Brock University”
“If you have **tried** (successful or not) to access formal support for emotional or mental health on campus, please describe any barriers you have encountered”.	Open-ended response
“If the following were available at Brock to prepare you for life after graduation, how likely would you be to access programs/information to help”,	“Find a family doctor in your community”, “Find other health professionals in your community (e.g. dentistry, occupational therapy, etc.)”, “Connect with mental health services in your community”, “Get supplemental health insurance (e.g. dental, prescriptions)”, “Apply for immigration/visa to remain in Canada or work/study abroad”, “Develop coping skills for managing stress”, “Develop healthy physical activity, eating, & other habits”, “Manage finances (e.g. student loans, debt, other budgeting)”, “Prepare to graduate with a peer support or mentorship program”
“Can you think of any other information, services, or programs that you would find helpful in preparing to graduate and/or the year after graduation?”	Open-ended response

## Appendix 6. Bivariate Pearson correlation coefficient for each mental health measure in Y 1. and Y 2. of the SHIFT survey

**Table:**

	Anxiety	Depression	Psychosocial Wellbeing	Perceived Stress
Anxiety	-	0.78**	0.08*	0.62**
Depression	0.78**	-	−0.02	0.62**
Psychosocial Wellbeing	0.08*	−0.02	-	0.32**
Perceived Stress	0.62**	0.62**	0.32**	-

*Correlation is significant at the *p* < 0.05 level.

**Correlation is significant at the *p* < .01 level.

## References

[cit0001] Aarø, L., Flisher, A., Kaaya, S., Onya, H., Francis, N., & Wubs, A. (2009, June). Parental education as an indicator of socioeconomic status: Improving quality of data by requiring consistency across measurement occasions. *Scandinavian Journal of Public Health*, 37(Suppl 2), 16–27. 10.1177/1403494808086917 19493978

[cit0002] Arnett, J. J. (2003). Conceptions of the transition to adulthood among emerging adults in American ethnic groups. *New Directions for Child and Adolescent Development*, 2003(100), 63–76. 10.1002/cd.75 12955983

[cit0003] Arnett, J. J. (2007). Emerging adulthood: What is it, and what is it good for? *Child Development Perspectives*, 1(2), 68–73. 10.1111/j.1750-8606.2007.00016.x

[cit0004] Arnett, J. J. (2015). *The oxford handbook of emerging adulthood*. Oxford University Press. 10.1093/oxfordhb/9780199795574.001.0001

[cit0005] Arnett, J. J., Žukauskienė, R., & Sugimura, K. (2014, December). The new life stage of emerging adulthood at ages 18–29 years: Implications for mental health. *The Lancet Psychiatry*, 1(7), 569–576. 10.1016/S2215-0366(14)00080-7 26361316

[cit0006] Baik, C., Larcombe, W., & Brooker, A. (2019, June). How universities can enhance student mental wellbeing: The student perspective. *Higher Education Research & Development*, 38(4), 674–687. 10.1080/07294360.2019.1576596

[cit0007] Barkham, M., Broglia, E., Dufour, G., Fudge, M., Knowles, L., Percy, A., Turner, A., & Williams, C. (2019, December). Towards an evidence‐base for student wellbeing and mental health: Definitions, developmental transitions and data sets. *Counselling & Psychotherapy Research*, 19(4), 351–357. 10.1002/capr.12227

[cit0008] Cage, E., Jones, E., Ryan, G., Hughes, G., & Spanner, L. (2021, September). Student mental health and transitions into, through and out of university: Student and staff perspectives. *Journal of Further and Higher Education*, 45(8), 1076–1089. 10.1080/0309877X.2021.1875203

[cit0009] Carver, J., Cappelli, M., Davidson, S. (2015). Taking the next step forward. Mental Health Commission of Canada, Retrieved May 3, 2022: https://www.mentalhealthcommission.ca/wp-content/uploads/drupal/Taking%252520the%252520Next%252520Step%252520Forward_0.pdf

[cit0010] Chaiton, M., Thorburn, R., Sutton, M., & Feng, P. (2023, March). LGBTQ2S+ youth perspectives on mental healthcare provider bias, standards of care, and accountability. *Youth*, 3(1), 93–106. 10.3390/youth3010006

[cit0011] Chen, S., Chen, H. (2010). Encyclopedia of Research design. SAGE Publications, Inc, doi:10.4135/9781412961288 [Internet]. Retrieved April 20, 2023. Available from: https://methods.sagepub.com/reference/encyc-of-research-design/n59.xml

[cit0012] Chrikov, I., Soria, K. M., Horgos, B., Jones-White, D. (2020). Undergraduate and graduate students’ mental health during the COVID-19 pandemic [Internet]. SERU Consortium, University of California - Berkeley and University of Minnesota, July Retrieved July 29, 2022: http://conservancy.umn.edu/handle/11299/215271

[cit0013] Coakley, K. E., Le, H., Silva, S. R., & Wilks, A. (2021, May). Anxiety is associated with appetitive traits in university students during the COVID-19 pandemic. *Nutrition journal*, 20(1), 45. 10.1186/s12937-021-00701-9 33985515 PMC8118620

[cit0014] Cohen, S. Perceived stress in a probability sample of the United States. [Internet]. Retrieved June 29, 2022: https://www.cmu.edu/dietrich/psychology/stress-immunity-disease-lab/scales/pdf/cohen,-s.–williamson,-g.-1988.pdf

[cit0015] Crumb, L., Crowe, A., Averett, P., Harris, J. A., & Dart, C. “Look like you have it together”: Examining mental Illness stigma and help seeking among diverse emerging adults. *Emerg Adulthood* 9(6), 702–711. 10.1177/2167696819852563

[cit0016] Currie, C. E., Elton, R. A., Todd, J., & Platt, S. (1997, September). Indicators of socioeconomic status for adolescents: The WHO health behaviour in school-aged children survey. *Health Education Research*, 12(3), 385–397. 10.1093/her/12.3.385 10174221

[cit0017] Czeisler, M. É., Lane, R. I., Petrosky, E., Wiley, J. F., Christensen, A., Njai, R., Weaver, M. D., Robbins, R., Facer-Childs, E. R., Barger, L. K., Czeisler, C. A., Howard, M. E., & Rajaratnam, S. M. W. (2020). Mental health, Substance use, and Suicidal Ideation during the COVID-19 pandemic — United States, June 24–30, 2020. *MMWR Morbidity and Mortality Weekly Report*, 69(32), 1049–1057. 10.15585/mmwr.mm6932a1 32790653 PMC7440121

[cit0018] Diener, E., Wirtz, D., Biswas-Diener, R., Tov, W., Kim-Prieto, C., Won, C. D., & Oishi, S. (2009). New measures of wellbeing. In *Assessing wellbeing: The collected works of Ed Diener*. Springer Science + Business Media, Social indicators research series (pp. 247–266) 10.1007/978-90-481-2354-4_12.

[cit0019] Doherty, D. T., & Kartalova O’Doherty, Y. (2010, February). Gender and self-reported mental health problems: Predictors of help seeking from a general practitioner. *British Journal of Health Psychology*, 15(1), 213–228. 10.1348/135910709X457423 19527564 PMC2845878

[cit0020] Domènech-Abella, J., Mundó, J., Miret, M., Ayuso-Mateos, J. L., Sánchez-Niubò, A., Abduljabbar, A. S., Haro, J. M., & Olaya, B. (2021, January). From childhood financial hardship to late-life depression: Socioeconomic pathways. *Aging & Mental Health*, 25(1), 86–93. 10.1080/13607863.2019.1671313 31597461

[cit0021] Drapeau, A., Boyer, R., & Diallo, F. B. (2011, October). Discrepancies between survey and administrative data on the use of mental health services in the general population: Findings from a study conducted in Québec. *BMC Public Health*, 11(1), 837. 10.1186/1471-2458-11-837 22040030 PMC3233633

[cit0022] Eisenberg, D., Gollust, S. E., Golberstein, E., & Hefner, J. L. (2007). Prevalence and correlates of depression, anxiety, and suicidality among university students. *The American Journal of Orthopsychiatry*, 77(4), 534–542. 10.1037/0002-9432.77.4.534 18194033

[cit0023] Eisenberg, D., Hunt, J., & Speer, N. (2012, August). Help seeking for mental health on college campuses: Review of evidence and next steps for research and practice. *Harvard Review of Psychiatry*, 20(4), 222–232. 10.3109/10673229.2012.712839 22894731

[cit0024] Eisenberg, D., Hunt, J., & Speer, N. (2013, January). Mental health in American colleges and universities: Variation across student subgroups and across campuses. *Journal of Nervous & Mental Disease*, 201(1), 60–67. 10.1097/NMD.0b013e31827ab077 23274298

[cit0025] Eisenberg, D., Lipson, S. K. (2019). 2018-2019 data report - healthy minds network [Internet]. Retrieved Mar 1, 2023, from https://healthymindsnetwork.org/wp-content/uploads/2019/09/HMS_national-2018-19.pdf

[cit0026] Fink, J. E. (2014). Flourishing: Exploring predictors of mental health within the college environment. *Journal of American College Health*, 62(6), 380–388. 10.1080/07448481.2014.917647 24779485

[cit0027] Fripp, J. A., & Carlson, R. G. (2017). Exploring the influence of attitude and stigma on participation of African American and Latino populations in mental health services. *Journal of Multicultural Counseling and Development*, 45(2), 80–94. 10.1002/jmcd.12066

[cit0028] Gondolf, E. W., & Williams, O. J. (2001). Culturally Focused Batterer Counseling for African American men. *Trauma, Violence & Abuse*, 2(4), 283–295. 10.1177/1524838001002004001

[cit0029] Grosemans, I., Hannes, K., Neyens, J., & Kyndt, E. (2020, July). Emerging adults embarking on their careers: Job and identity explorations in the transition to work. *Youth & Society*, 52(5), 795–819. 10.1177/0044118X18772695

[cit0030] Guan, N., Guariglia, A., Moore, P., Xu, F., Al-Janabi, H., & Magalhaes, P. V. D. S. (2022, February). Financial stress and depression in adults: A systematic review. *PLOS ONE*, 17(2), e0264041. 10.1371/journal.pone.0264041 35192652 PMC8863240

[cit0031] Hadlaczky, G., Hökby, S., Mkrtchian, A., Carli, V., & Wasserman, D. (2014, August). Mental health first aid is an effective public health intervention for improving knowledge, attitudes, and behaviour: A meta-analysis. *International Review of Psychiatry*, 26(4), 467–475. 10.3109/09540261.2014.924910 25137113

[cit0032] Hallett, R. E., Kezar, A., Perez, R. J., & Kitchen, J. A. (2020, March). A typology of college transition and support programs: Situating a 2-year comprehensive college transition program within college access. *The American Behavioral Scientist*, 64(3), 230–252. 10.1177/0002764219869410

[cit0033] Hone, L., Jarden, A., & Schofield, G. (2014). Psychometric properties of the flourishing scale in a New Zealand sample. *Social Indicators Research*, 119(2), 1031–1045. 10.1007/s11205-013-0501-x

[cit0034] House, L. A., Neal, C., & Kolb, J. (2020, April). Supporting the mental health needs of first generation college students. *Journal of College Student Psychotherapy*, 34(2), 157–167. 10.1080/87568225.2019.1578940

[cit0035] Howell, A., & Buro, K. (2015). Measuring and predicting student wellbeing: Further evidence in support of the flourishing scale and the scale of positive and negative experiences. *Soc Indic Res Int Interdiscip J Qual–Life Meas*, 121(3), 1. 10.1007/s11205-014-0663-1

[cit0036] Hyun, J. K., Quinn, B. C., Madon, T., & Lustig, S. (2006). Graduate student mental health: Needs assessment and utilization of counseling services. *Journal of College Student Development*, 47(3), 247–266. 10.1353/csd.2006.0030

[cit0037] Jenkins, E. K., Slemon, A., Richardson, C., Pumarino, J., McAuliffe, C., Thomson, K. C., Goodyear, T., Daly, Z., McGuinness, L., & Gadermann, A. (2022). Mental health inequities Amid the COVID-19 pandemic: Findings from three Rounds of a cross-sectional Monitoring survey of Canadian adults. *International Journal of Public Health*. 10.3389/ijph.2022.1604685 PMC9349347 35936999

[cit0038] Jin, Y., Zhu, D., & He, P. (2020, March 1). Social causation or social selection? The longitudinal interrelationship between poverty and depressive symptoms in China. *Social Science & Medicine*, 249, 112848. 10.1016/j.socscimed.2020.112848 32087488

[cit0039] Jorm, A. F. (2012, April). Mental health literacy: Empowering the community to take action for better mental health. *The American Psychologist*, 67(3), 231–243. 10.1037/a0025957 22040221

[cit0040] Keyes, C. L. M. (2005, June). Mental Illness and/or mental health? Investigating axioms of the complete state model of health. *Journal of Consulting & Clinical Psychology*, 73(3), 539–548. 10.1037/0022-006X.73.3.539 15982151

[cit0041] Keyes, C. L. M. (2007). Promoting and protecting mental health as flourishing: A complementary strategy for improving national mental health. *American Psychologist*, 62(2), 95–108. 10.1037/0003-066X.62.2.95 17324035

[cit0042] Keyes, C. L. M. (2009). The Black–White paradox in health: Flourishing in the face of social inequality and discrimination. *Journal of Personality*, 77(6), 1677–1706. 10.1111/j.1467-6494.2009.00597.x 19796064

[cit0043] Kirmayer, L. J., Narasiah, L., Munoz, M., Rashid, M., Ryder, A. G., Guzder, J., Hassan, G., Rousseau, C., & Pottie, K. (2011, September). Common mental health disorders in immigrants and refugees: General approach in primary care. *Canadian Medical Association Journal*, 183(12), E959–67. 10.1503/cmaj.090292 20603342 PMC3168672

[cit0044] Kirsch, D. J., Pinder-Amaker, S. L., Morse, C., Ellison, M. L., Doerfler, L. A., & Riba, M. B. (2014, December). Population-based initiatives in college mental health: Students helping students to overcome obstacles. *Current Psychiatry Reports*, 16(12), 525. 10.1007/s11920-014-0525-1 25308393

[cit0045] Kreniske, P., Mellins, C. A., Shea, E., Walsh, K., Wall, M., Santelli, J. S., Reardon, L., Khan, S., Hwei, T., & Hirsch, J. S. (2022, September). Associations between low-household income and first-generation status with college student belonging, mental health, and well-being. *Emerg Adulthood*, 11(3), 710–720. 10.1177/21676968221124649 40800720 PMC12341491

[cit0046] Krieger, N. (1990, January). Racial and gender discrimination: Risk factors for high blood pressure? *Social Science & Medicine*, 30(12), 1273–1281. 10.1016/0277-9536(90)90307-E 2367873

[cit0047] Kroenke, K., Spitzer, R. L., & Williams, J. B. W. (2001, September). The PHQ-9: Validity of a brief depression severity measure. *Journal of General Internal Medicine*, 16(9), 606–613. 10.1046/j.1525-1497.2001.016009606.x 11556941 PMC1495268

[cit0048] Linden, B., & Jurdi-Hage, R. (2017). Examining the predictors of mental health outcomes among undergraduate postsecondary students in Canada. *Journal of Social, Behavioral and Health Sciences*, 11(1). 10.5590/JSBHS.2017.11.1.01

[cit0049] Lindsey, M. A., Chambers, K., Pohle, C., Beall, P., & Lucksted, A. (2013, January). Understanding the behavioral determinants of mental health service use by urban, under-resourced Black Youth: Adolescent and caregiver perspectives. *Journal of Child & Family Studies*, 22(1), 107–121. 10.1007/s10826-012-9668-z 23355768 PMC3551580

[cit0050] Luo, Y., & Waite, L. J. (2005). The impact of childhood and adult SES on physical, mental, and cognitive wellbeing in later life. *The Journals of Gerontology: Series B*, 60(2), S93–S101. 10.1093/geronb/60.2.S93 PMC2505177 15746030

[cit0051] MacLeod, K. B., & Brownlie, E. B. (2014, July). Mental health and transitions from adolescence to emerging adulthood: Developmental and diversity considerations. *Canadian Journal of Community Mental Health*, 33(1), 77–86. 10.7870/cjcmh-2014-007

[cit0052] Manea, L., Gilbody, S., & McMillan, D. (2012). Optimal cut-off score for diagnosing depression with the Patient Health Questionnaire (PHQ-9): A meta-analysis. *CMAJ*, 184(3), E191–E196. 10.1503/cmaj.110829 22184363 PMC3281183

[cit0053] McCloud, T., & Bann, D. (2019). Financial stress and mental health among higher education students in the UK up to 2018: Rapid review of evidence. *Journal of Epidemiology & Community Health*, 73(10), 977–984. 10.1136/jech-2019-212154 31406015 PMC6817692

[cit0054] Meyer, I. P. (2003, September). Social stress, and mental health in Lesbian, Gay, and Bisexual populations: Conceptual issues and research evidence. *Psychological Bulletin*, 129(5), 674–697. 10.1037/0033-2909.129.5.674 12956539 PMC2072932

[cit0055] Mori, S. C. (2000). Addressing the mental health concerns of International students. *Journal of Counseling and Development: JCD*, 78(2), 137–144. 10.1002/j.1556-6676.2000.tb02571.x

[cit0056] Mossman, S. A., Luft, M. J., Schroeder, H. K., Varney, S. T., Fleck, D. E., & Barzman, D. H. (2018). The Generalized anxiety disorder 7-item (GAD-7) scale in adolescents with generalized anxiety disorder: Signal detection and validation, 14. PMC5765270 29069107

[cit0057] National Alliance on Mental Illness. African American community mental health fact sheet [Internet]. Retrieved February 28, 2023, from https://namihowardcounty.org/wp-content/uploads/sites/174/2015/01/AfricanAmericanMentalHealthFactSheet.pdf

[cit0058] OECD. (2013). The territorial impact of COVID-19: Managing the crisis across levels of government [Internet]. Retrieved April 26, 2023, from https://www.oecd.org/coronavirus/policy-responses/the-territorial-impact-of-covid-19-managing-the-crisis-across-levels-of-government-d3e314e1/

[cit0059] Oke, J., Akinkunmi, W., & Etebefia, S. (2022, September 26). Use of correlation, tolerance and variance inflation factor for multicollinearity test. 7.

[cit0060] Pancer, S. M., Hunsberger, B., Pratt, M. W., & Alisat, S. (2000, January). Cognitive complexity of expectations and adjustment to university in the first year. *Journal of Adolescent Research*, 15(1), 38–57. 10.1177/0743558400151003

[cit0061] Paul, E. L., & Brier, S. (2001). Friendsickness in the transition to college: Precollege predictors and college adjustment correlates. *Journal of Counseling and Development: JCD*, 79(1), 77–89. 10.1002/j.1556-6676.2001.tb01946.x

[cit0062] Popping, R. (2015, October). Analyzing open-ended questions by means of text analysis Procedures. *Bull Sociol Methodol Méthodologie Sociol*, 128(1), 23–39. 10.1177/0759106315597389

[cit0063] Richardson, T., Elliott, P., Roberts, R., & Jansen, M. (2017). A longitudinal study of financial difficulties and mental health in a National sample of British undergraduate students. *Community Mental Health Journal*, 53(3). 344–352. 10.1007/s10597-016-0052-0 27473685 PMC5337246

[cit0064] Romano, I., Ferro, M. A., Patte, K. A., Diener, E., & Leatherdale, S. T. (2020, November). Measurement invariance of the flourishing scale among a large sample of Canadian adolescents. *International Journal of Environmental Research and Public Health*, 17(21), 7800. 10.3390/ijerph17217800 33113772 PMC7663739

[cit0065] Ryff, C. D., Keyes, C. L. M., & Hughes, D. L. (2003). Status inequalities, perceived discrimination, and eudaimonic well-being: Do the challenges of minority life hone purpose and growth? *Journal of Health & Social Behavior*, 44(3), 275–291. 10.2307/1519779 14582308

[cit0066] Ryu, S., & Fan, L. (2022, February). The relationship between financial worries and psychological distress among U.S. adults. *Journal of Family and Economic Issues*, 44(1), 16–33. 10.1007/s10834-022-09820-9 35125855 PMC8806009

[cit0067] Sainani, K. L. (2012, December). Dealing with non-normal data. *PM&R*, 4(12), 1001–1005. 10.1016/j.pmrj.2012.10.013 23245662

[cit0068] Schroeder, M. A., Lander, J., & Levine-Silverman, S. (1990, April). Diagnosing and dealing with multicollinearity. *Western Journal of Nursing Research*, 12(2), 175–187. 10.1177/019394599001200204 2321373

[cit0069] Silver, B. R., & Roksa, J. (2017, July). Navigating uncertainty and responsibility: Understanding inequality in the senior-year transition. *Journal of Student Affairs Research and Practice*, 54(3), 248–260. 10.1080/19496591.2017.1331851

[cit0070] Sontag-Padilla, L., Dunbar, M. S., Ye, F., Kase, C., Fein, R., Abelson, S., Seelam, R., & Stein, B. D. (2018, July). Strengthening college students’ mental health knowledge, awareness, and helping behaviors: The impact of active minds, a peer mental health organization. *Journal of the American Academy of Child & Adolescent Psychiatry*, 57(7), 500–507. 10.1016/j.jaac.2018.03.019 29960695

[cit0071] Spitzer, R. L., Kroenke, K., Williams, J. B. W., & Löwe, B. (2006, May). A brief measure for assessing generalized anxiety disorder: The GAD-7. *Archives of Internal Medicine*, 166(10), 1092. 10.1001/archinte.166.10.1092 16717171

[cit0072] Stansfeld, S. A., Clark, C., Rodgers, B., Caldwell, T., & Power, C. (2008, February). Childhood and adulthood socio-economic position and midlife depressive and anxiety disorders. *The British Journal of Psychiatry: The Journal of Mental Science*, 192(2), 152–153. 10.1192/bjp.bp.107.043208 18245036

[cit0073] Stepped care 2.0. (2022). Mental health Commission of Canada [Internet]. Mental Health Commission of Canada. Retrieved Mar 1, 2023, from https://mentalhealthcommission.ca/what-we-do/access/stepped-care-2-0/

[cit0074] Syed, M., & Race, M. L. (2013). Ethnicity, and emerging adulthood: Retrospect and prospects. *Emerging Adulthood*, 1(2), 83–95. 10.1177/2167696813480503

[cit0075] Tellinghuisen, J. (2008). Least squares with non-normal data: Estimating experimental variance functions. *The Analyst*, 133(2), 161–166. 10.1039/B708709H 18227936

[cit0076] Toomey, R. B., Ryan, C., Diaz, R. M., Card, N. A., & Russell, S. T. (2010, November). Gender-nonconforming lesbian, gay, bisexual, and transgender youth: School victimization and young adult psychosocial adjustment. *Developmental Psychology*, 46(6), 1580–1589. 10.1037/a0020705 20822214

[cit0077] Turner, H. (2023). Gender and social support: Taking the bad with the good? *SpringerLink* Internet. https://link.springer.com/article/10.1007/BF01420800

[cit0078] Wallach, S., Garner, A., Howell, S., Adamson, T., Baral, S., & Beyrer, C. (2020, December). Address Exacerbated health disparities and Risks to LGBTQ+ individuals during COVID-19. *Health and Human Rights*, 22(2), 313. 33390717 PMC7762918

[cit0079] Warttig, S. L., Forshaw, M. J., South, J., & White, A. K. (2013). New, normative, English-sample data for the Short Form perceived stress scale (PSS-4). *Journal of Health Psychology*, 18(12), 1617–1628. 10.1177/1359105313508346 24155195

[cit0080] Wickrama, K. A. S., Noh, S., & Bryant, C. M. (2005). Racial differences in adolescent distress: Differential effects of the family and community for Blacks and Whites. *Journal of Community Psychology*, 33(3), 261–282. 10.1002/jcop.20053

[cit0081] Wierzbicki, M., & Pekarik, G. (1993). A meta-analysis of psychotherapy dropout. *Professional Psychology: Research and Practice*, 24(2), 190–195. 10.1037/0735-7028.24.2.190

[cit0082] Wiljer, D., Abi-Jaoude, A., Johnson, A., Ferguson, G., Sanches, M., Levinson, A., Robb, J., Heffernan, O., Herzog, T., Chaim, G., Cleverley, K., Eysenbach, G., Henderson, J., S Hoch, J., Hollenberg, E., Jiang, H., Isaranuwatchai, W., Law, M. & Tripp, T. (2016, November). Enhancing self-efficacy for help-seeking among transition-aged youth in postsecondary settings with mental health and/or substance use concerns, using crowd-sourced online and mobile technologies: The thought spot protocol. *JMIR Research Protocols*, 5(4), e201. 10.2196/resprot.6446 27815232 PMC5116103

[cit0083] Xiong, J., Lipsitz, O., Nasri, F., Lui, L. M. W., Gill, H., Phan, L., Chen-Li, D., Iacobucci, M., Ho, R., Majeed, A., & McIntyre, R. S. (2020, December 1). Impact of COVID-19 pandemic on mental health in the general population: A systematic review. *Journal of Affective Disorders*, 277, 55–64. 10.1016/j.jad.2020.08.001 32799105 PMC7413844

