# Correction

**DOI:** 10.1080/28324765.2025.2457875

**Published:** 2025-01-28

**Article title**: Final-year university students’ mental health and access to support as they prepared to graduate

**Authors**: Megan J. Magier, Madelyn Law, Sarah Pennisi, Tanya Martini, Markus J Duncan, Hussain Chattha and Karen A Patte

**Journal**: Cogent Mental Health

**DOI**: 10.1080/28324765.2023.2252918

This article was originally published with an error in the text. To rectify this, the following additional content has now been included at the beginning of the Data Collection section.

Before correction: The initial email invitation with a link to the survey hosted on Qualtrics was sent on 19 April 2021, in the first year, and on 18 April 2022, in the second year, with two follow-up reminder emails both years. The surveys remained open for 3-week data collection periods. Participants were able to enter an optional draw to win one of ten $50 gift cards.

After correction: An invitation letter and subsequent follow-up email reminders included a link to the Information and Consent page on Qualtrics. Participants were required to provide consent by clicking a checkbox on the online Information and Consent page before proceeding to the survey. The initial email invitation with a link to the survey hosted on Qualtrics was sent on 19 April 2021, in the first year, and on 18 April 2022, in the second year, with two follow-up reminder emails both years. The surveys remained open for 3-week data collection periods. Participants were able to enter an optional draw to win one of ten $50 gift cards.

